# Response: Commentary: Belatacept Does Not Inhibit Follicular T Cell-Dependent B-Cell Differentiation in Kidney Transplantation

**DOI:** 10.3389/fimmu.2018.00466

**Published:** 2018-03-08

**Authors:** Carla C. Baan, Gretchen N. de Graav, Willem Weimar, Dennis A. Hesselink

**Affiliations:** ^1^Section Nephrology and Transplantation, Department Internal Medicine, The Rotterdam Transplant Group, Erasmus MC, University Medical Center, Rotterdam, Netherlands

**Keywords:** belatacept, kidney transplantation, costimulation blockade, follicular helper T cells, rejection

The commentary by Schroder et al. on our report “*Belatacept Does Not Inhibit Follicular T Cell-Dependent B-Cell Differentiation in Kidney Transplantation*,” challenges our findings and conclusions by questioning the methodology, definitions and interpretation of the results (1). We appreciate the comments and acknowledge that discussion about the mechanistic basis of the rejection response will advance our understanding of this process and may improve outcomes after transplantation, the goal of the transplant community.

After transplantation, a cellular infiltrate in the transplanted organ consisting of both T and B cells is a hallmark of the anti-donor rejection response. In our study, we questioned whether a subset of T cells, the CXCR5^+^CD4^+^ T follicular (Tfh) cells, provides help to B cells in immunosuppressed kidney transplant patients (2–4). We examined Tfh–B cell interactions in patients treated with either belatacept- or tacrolimus-based immunosuppression [a CTLA4-immunoglobulin (Ig) and an inhibitor of the CD28-CD80/86 pathway, and an inhibitor of the calcineurin pathway, respectively] (2, 4). The capacity of Tfh cells to provide help to B cells depends on costimulatory molecules such as ICOS, PD-1, CD28, and the cytokine IL-21 (3).

In brief, three arguments were put forward as to why we should have been more cautious in our conclusions (1). The first argument is that we investigated the isolated effects of belatacept and that our study lacked unmodified controls; second, that Tfh–B cell interactions were studied in peripheral blood samples; and third, the definition we used to define Tfh cells.

We believe that Schroder et al. possibly overlooked some of the details of our study. As summarized in Figure 1, different comparisons were made, including the unmodified immune response (2). We agree that isolated immunosuppressive drug effect studies may not reflect the *in vivo* situation in patients and for this reason we included data labeled “*in vivo* drug studies.” These demonstrated the *in vivo* effect of the immunosuppressive treatment in our patient group. The capacity for Tfh cell generation, activation, and the ability to mediate B cell differentiation and Ig production, was tested in patient samples rechallenged *in vitro* with donor antigen. These experiments demonstrated that peripheral Tfh cells remained functional. In this respect, our opinion differs from that of Schroder et al., who suggest a long-lasting effect of belatacept and tacrolimus on isolated Tfh and B cells (1). To be certain that the effect of drugs can be measured at all in our system, belatacept and tacrolimus were added to the interaction of donor antigen Tfh–B cells, which resulted in a median inhibition of 33 and 55%, respectively [*in vitro* drug experiments; Figure 5 (2)]. These findings led us to the conclusion that the immunosuppressive drugs studied—including belatacept—have a limited effect. We are of course aware that combination therapy, including mycophenolate mofetil and steroids, may influence Tfh–B cell cross talk *in vivo*. The unique setting of our study, i.e., donor antigen-specific activation of the Tfh–B cell interaction in peripheral blood cell samples from kidney transplant patients, can result in different outcomes from isolated Tfh cell studies [Figure S2 in Supplementary Material (2)] that certainly do not reflect the real-world situation in human transplant recipients, let alone from studies based on cells from healthy blood bank donors or *in vitro* generated Tfh cells (1, 2). A study by Badell et al. has reported clear-cut effects of CTLA4-Ig in the prevention of Tfh cell-mediated humoral immune activity in a murine skin transplant model (2, 5). As acknowledged by Ford [co-author of Schroder et al. (1)], findings from transplant models using inbred animals are difficult to extrapolate to transplantation in patients with an adult immune system (6). Substantial differences exist in terms of the basal immune state of these mice in comparison to patients which can contribute to the findings in studies (2, 5–7).

The second limitation ascribed to our study is that a study of the secondary lymphoid organs, where most Tfh–B cell interactions occur, is lacking (8). This reflects the conventional opinion that immune responses occur in these organs. In transplantation, however, Tfh–B cell interactions also occur in the transplanted organ itself. We demonstrated the presence of Bcl6+ (Tfh-transcription factor) and IL-21^+^ T cells in kidney allograft biopsies that colocalized with B cells and Igs during rejection (9, 10). Our biopsy studies further revealed that in belatacept treated patients the target molecule, CD86, was still expressed by mononuclear cells, which can be explained by the small distribution volume of belatacept (11, 12). We agree that unraveling the function of Tfh and B cells residing in secondary lymphoid organs will provide mechanistic insights into the pathways involved in alloreactivity, which will, in turn, help us to develop better pharmacologic inhibitors to control cellular and antibody-mediated allograft rejection (13, 14). However, because of ethical considerations, this can only be studied in animal transplant models.

Finally, with regard to the comment about the definition of peripheral Tfh cells: the role of Tfh cell humoral immunity has been recognized in the field of human immunology, where it has been shown that circulating CXCR5^+^CD4^+^ T cells are the counterparts of germinal center Tfh cells (3, 15). These Tfh cells may differ from lymph node Tfh cells since peripheral Tfh cells express ICOS and PD-1 at low levels. However, in an allogeneic setting circulating CXCR5^+^CD4^+^ T cells have the capacity to provide help to B cells (16). Nevertheless, the definition of Tfh cells by CXCR5 is only an approximation, and some fine tuning is required.

Over the past few years, we have made major progress in understanding how immunosuppressive drugs act in patients through the study of blood and biopsy material. In belatacept-treated patients, the incomplete blockade of costimulatory pathways and redundancy in the immune system may contribute to the apparent lack of efficacy when tested *in vitro* and in patients (2, 4, 17). Direct translation of findings in different models is difficult—if not impossible—because the test model influences the outcome and consequently, the conclusions. We have to work in collaboration to find improved models that will, eventually, help us find solutions for the current challenges such as effective treatment of antibody-mediated rejection.

## Author Contributions

CB, GG, WW, and DH participated in writing this text.

## Conflict of Interest Statement

The authors declare that the research was conducted in the absence of any commercial or financial relationships that could be construed as a potential conflict of interest.
